# *Psychrobacillus syltrankelensis* sp. nov., a new species isolated from soil in the North Caucasus, Russia

**DOI:** 10.1128/spectrum.02919-25

**Published:** 2026-04-20

**Authors:** Maria N. Romanenko, Anton E. Shikov, Fedor M. Shmatov, Iuliia A. Savina, Mikhail V. Belousov, Alexei Solovchenko, Olga Chivkunova, Grigoriy K. Savelev, Irina G. Kuznetsova, Denis S. Karlov, Anton A. Nizhnikov, Kirill S. Antonets

**Affiliations:** 1 All-Russia Research Institute for Agricultural Microbiology 117473, St. Petersburg, Russia; 2 Faculty of Biology, St. Petersburg State University 48544, St. Petersburg, Russia; 3 Department of Bioengineering, Faculty of Biology, Moscow State University 64935, Moscow, Russia; Instituto de Ecología, A.C. (INECOL), Pátzcuaro, Michoacán, Mexico

**Keywords:** *Psychrobacillus syltrankelensis*, North Caucasus soil, genome assembly, BGC, streptozotocin, PANC-1, cytotoxicity, chemotaxonomy, carbon source utilization

## Abstract

Members of the genus *Psychrobacillus* are widely distributed across diverse environments, and novel representatives continue to be identified. Here, we describe the strain nc5.1^T^, isolated from soil near Lake Syltran-Kel in the Kabardino-Balkarian Republic, Russia. Phylogenomic analysis indicated that *P. glaciei* PB01^T^ was its closest relative, placing the isolate within the genus *Psychrobacillus*. The average nucleotide identity (84.3%) and digital DNA–DNA hybridization (31%) values compared with PB01^T^ suggested that the isolate represents a novel species. The strain nc5.1^T^ was a Gram-negative, motile, rod-shaped bacterium forming circular, smooth, beige-brown colonies. No morphologically detectable endospores were observed under sporulation-inducing conditions. Optimal growth conditions were pH 6.5, 0.5% NaCl, and 30°C; however, a minor but reproducible viable fraction persisted after exposure to 80°C. Biochemical profiling revealed that the strain assimilated 46 carbon sources spanning carbohydrates, amino acids, and organic acids and exhibited growth in the presence of aztreonam, nalidixic acid, and potassium tellurite. The dominant cellular fatty acids were C_14:1_ (52.8%), C_14:0_ (16.0%), and C_16:0_ (7.7%), which distinguished the isolate from *P. glaciei*. Genomic analysis identified biosynthetic gene clusters associated with the synthesis of iturin and a streptozotocin-like moiety, suggesting the capacity for biosynthesis of secondary metabolites with beneficial properties. MTT assays showed that culture supernatants did not reduce mammalian cell viability but, conversely, moderately stimulated the proliferation of PANC-1 cells. Together, the phenotypic, chemotaxonomic, and genomic data indicate that strain nc5.1^T^ represents a novel species with the proposed name *Psychrobacillus syltrankelensis* sp. nov. The type strain is nc5.1^T^ (=RCAM 07221).

**IMPORTANCE:**

The discovery of the *P. syltrankelensis* species extends our understanding of diversity within *Bacillaceae* and reveals traits that challenge the established views of the family. Based on genomic thresholds, nc5.1ᵀ clearly represents a novel species while also displaying atypical morphological features, such as Gram-negative staining and absence of sporulation. Despite the lack of morphologically detectable endospores, the strain retained viability after exposure to elevated temperatures, further emphasizing its unusual physiological profile. These characteristics underscore the limitations of morphology-based taxonomy and the necessity of genome-guided classification. Genome analysis revealed two adaptive groups within *Psychrobacillus*, distinguished by mutually exclusive biosynthetic clusters responsible for the synthesis of either iturin or bacillomycin D, with nc5.1ᵀ belonging to the iturin-producing lineage. The nc5.1ᵀ isolate also harbored a highly divergent streptozotocin-like locus, indicating unexplored secondary metabolism with potential biotechnological relevance. At the same time, culture supernatants unexpectedly stimulated the growth of human pancreatic carcinoma cells, underlining the complexity of functional predictions based solely on genomic data. Together, these findings highlight the importance of combining phenotypic, ecological, and genomic approaches for accurate species delineation and provide new insights into the hidden metabolic potential of *Psychrobacillus*.

## INTRODUCTION

The *Psychrobacillus* genus includes bacilliform spore-forming bacteria occupying a wide range of ecological niches (1, 2). It was accepted as a separate genus in 2010 after reclassification of the three species previously belonging to the *Bacillus* genus, namely *P. insolitus*, *P. psychrodurans*, and *P. psychrotolerans* (1). To date, 10 *Psychrobacillus* species are known, with the latest described unit *P. mangrovi* (3).

Distinct *Psychrobacillus* strains were isolated from various specific ecotopes, including glaciers (4), animal feces (5), soil (6), contaminated areas (7), plants (8, 9), etc. Predominantly, representatives of the species are psychrophiles, i.e., capable of withstanding low temperatures (2). Despite the genus remaining poorly studied both experimentally and genomically, the existing reports demonstrate that *Psychrobacillus* isolates could possess beneficial traits, such as the ability to degrade oil (7), production of bioemulsifier (6), endophytic properties (9), and plant growth-promoting activity (8).

At present, only 54 genomes are deposited in the NCBI RefSeq database (10), which highlights the taxonomic versatility of the genus, given a high proportion of distinct species within a relatively small data set. Therefore, employing comprehensive analysis is required to refine our understanding of the intricate taxonomy of the genus.

In this research, we identified the *Psychrobacillus* isolate nc5.1 inhabiting the Caucasus Mountains. This region is characterized by unequally distributed conditions in terms of both vertical and horizontal zonality, seasonal, and day-and-night changes (11–13). While little is known about the biodiversity of microorganisms from this region, metagenomic studies illustrate a visible heterogeneity in microbial communities belonging to diverse biomes. For instance, in mineral water aquifers, the phylum *Bacillota* accounted for almost 80% of the community (14), while the cave microbiome was dominated by Actinobacteria (15). The Caucasus region is a rich source of hidden bacterial diversity yet to be discovered. The strains forming novel taxonomic categories were isolated from the Caucasus, such as the genera of the *Thiothrix* (16) and *Thiocaldithrix* (17), or even a new order of *Anaerosomatales* (17).

To the best of our knowledge, the isolates of *Psychrobacillus* spp. have not been reported in the Caucasus. The isolate described in this research, by all criteria, including average nucleotide identity (ANI) and digital DNA-DNA hybridization (DDH), reaching 84.31% and 31%, respectively, relative to the closest type strain *P. glaciei* PB01^T^, constitutes a novel species termed *Psychrobacillus syltrankelensis* nc5.1^T^ sp. nov. In addition to genomic properties, it also differs substantially in terms of the chemical composition of membranes, morphology, and the spectrum of metabolic activities. Despite being close to *P. glaciei*, our strain lacks psychrotolerance, unlike other members of the genus. A combination of genomic, detailed morphological, and physiological descriptions of the *P. syltrankelensis* nc5.1^T^ sp. nov. presented here, therefore, assists in unraveling the taxonomic relationships within the whole genus.

## RESULTS

### Isolation of the type strain

Strain nc5.1 was isolated from a soil sample collected near Lake Syltran-Kel, Kabardino-Balkaria, Russia, and subsequently deposited in the Russian Collection of Agricultural Microorganisms (RCAM) at the All-Russia Research Institute for Agricultural Microbiology, Saint Petersburg, under the accession number RCAM 07,221 (https://rcam.arriam.ru/, accessed 30 August 2025). To obtain a detailed characterization of the strain, we first determined its optimal growth conditions, which were then applied for subsequent morphological and physiological analyzes.

### Optimal growth conditions of the strain

**Fig 1:**
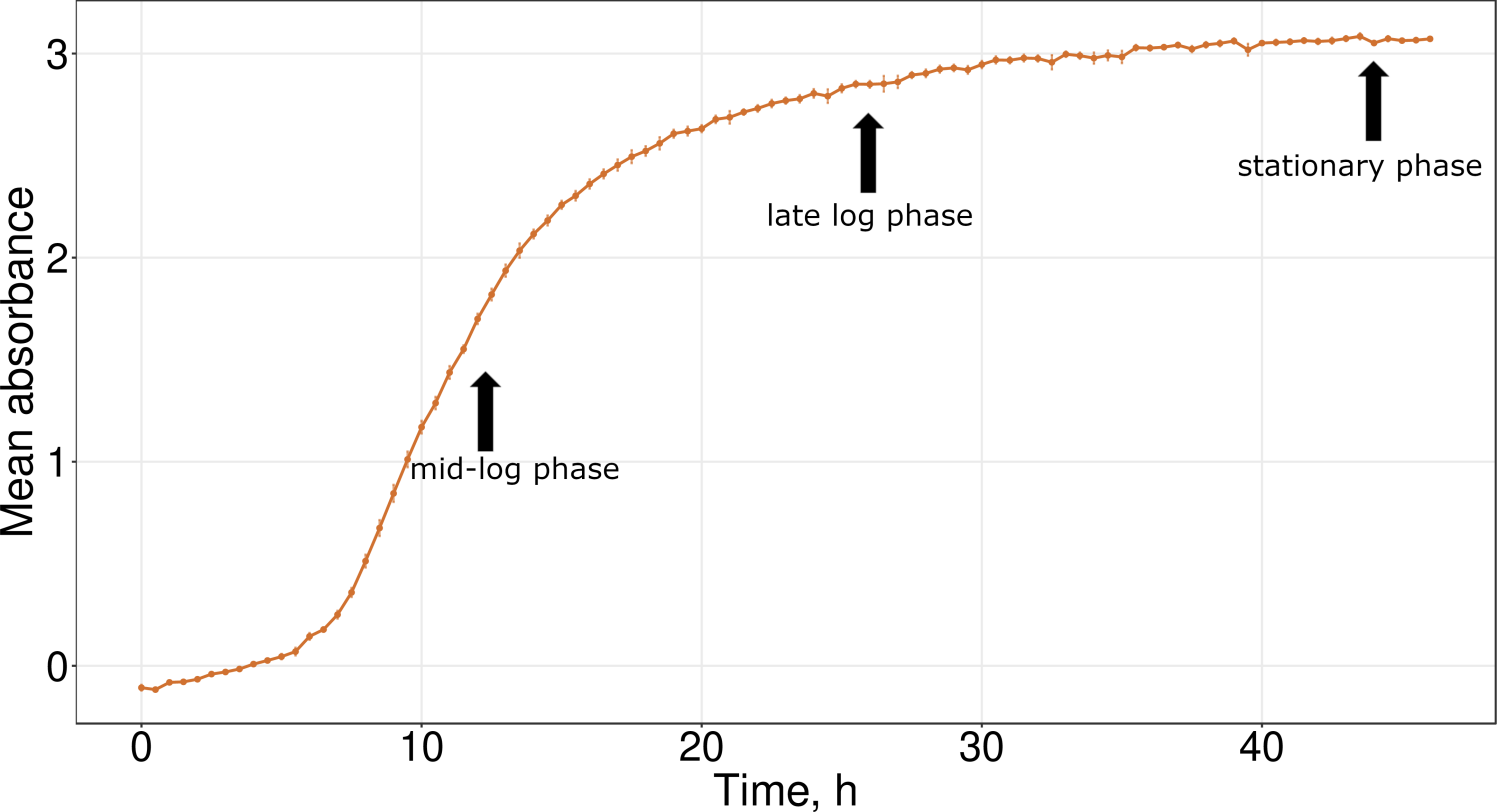
Growth curve of the nc5.1 strain. The vertical axis denotes the optical density of the bacterial culture at 600 nm. Uninoculated medium has been used as a blank sample. Black arrows denote different phases of bacterial growth: mid-log, late log, and stationary.

The strain tolerated a pH range of 6.0–8.0, NaCl concentrations from 0% to 1% (w/v), and temperatures between 20°C and 35°C. Optimal growth was observed at pH 6.5, 0.5% NaCl, and 30°C, which corresponded to the basic composition of the 2YT medium in terms of pH and salinity. At the optimal conditions, the bacterial culture reaches the late-log phase after 26 h of incubation and the stationary phase after 44 h (Fig. 1).

### Morphological characteristics

**Fig 2:**
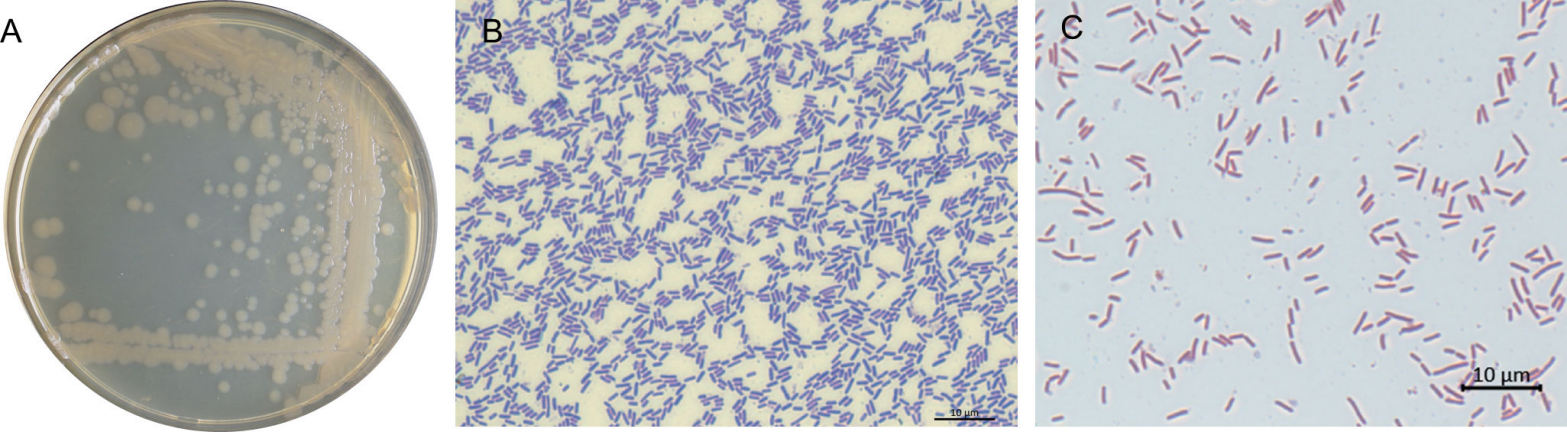
Morphological characteristics of the nc5.1 strain. (**A**) Colony morphology after 24 h of cultivation on 2TY medium. (**B**) Cell morphology on the sixth day of growth stained with Coomassie Brilliant Blue. (**C**) Gram-stained cells showing a Gram-negative reaction. Scale bar = 500 µm.

After 24 h of cultivation under optimal growth conditions, colonies of the strain nc5.1 measured 2–8 mm in diameter, were circular with smooth margins, and exhibited a flat profile with a homogeneous internal structure. The colony surface was smooth, shiny, and transparent, with a brownish-beige pigmentation (Fig. 2A). Light microscopy of Coomassie Brilliant Blue–stained preparations revealed rod-shaped cells that did not form spores (Fig. 2B).

**Fig 3:**
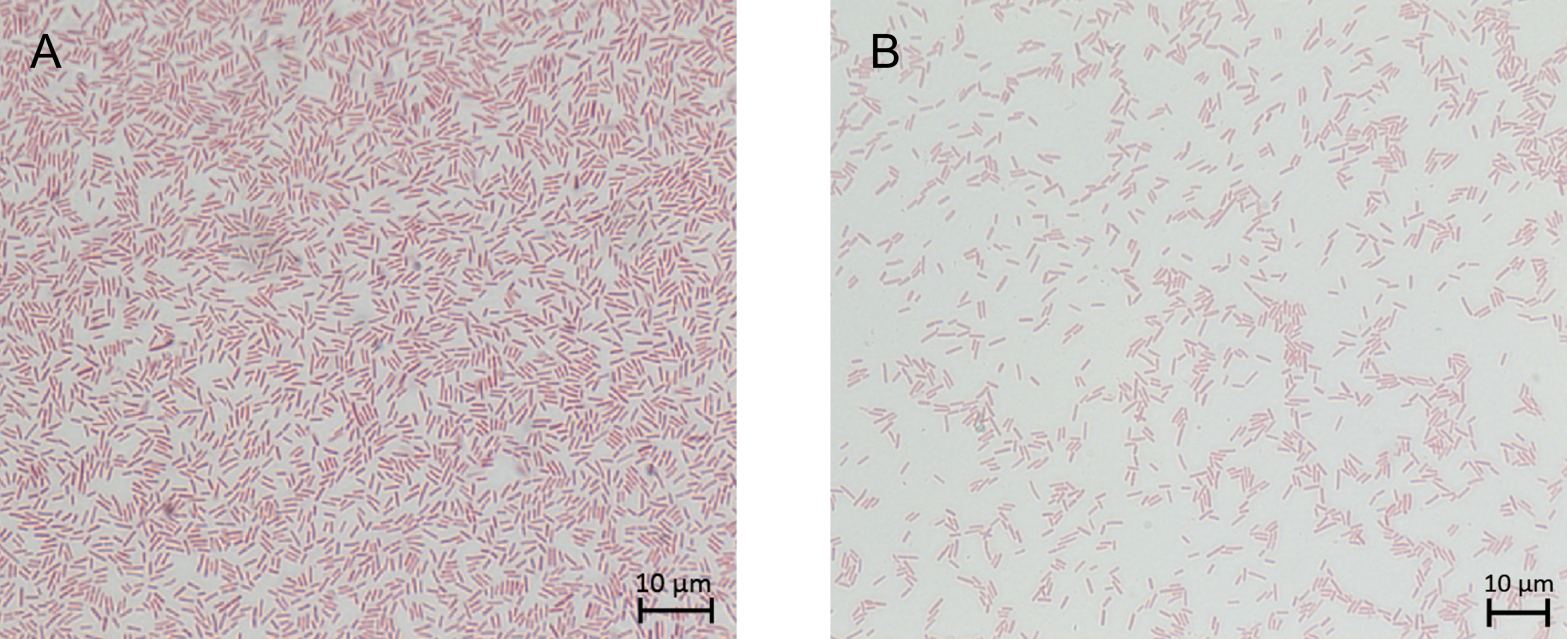
Schaeffer–Fulton endospore staining of the strain nc5.1. (**A**) Cells after 120 h of cultivation on T3 agar medium. (**B**) Cells after 120 h of cultivation in Schaeffer’s sporulation medium.

Following Schaeffer–Fulton staining, uniform red staining of rod-shaped bacterial cells was observed, with no green-stained endospores detected under the tested conditions. This pattern was observed in samples cultivated on T3 agar medium (18) (Fig. 3A) and in those grown in Schaeffer’s sporulation medium (19) even after 120 h of cultivation (Fig. 3B).

**Fig 4:**
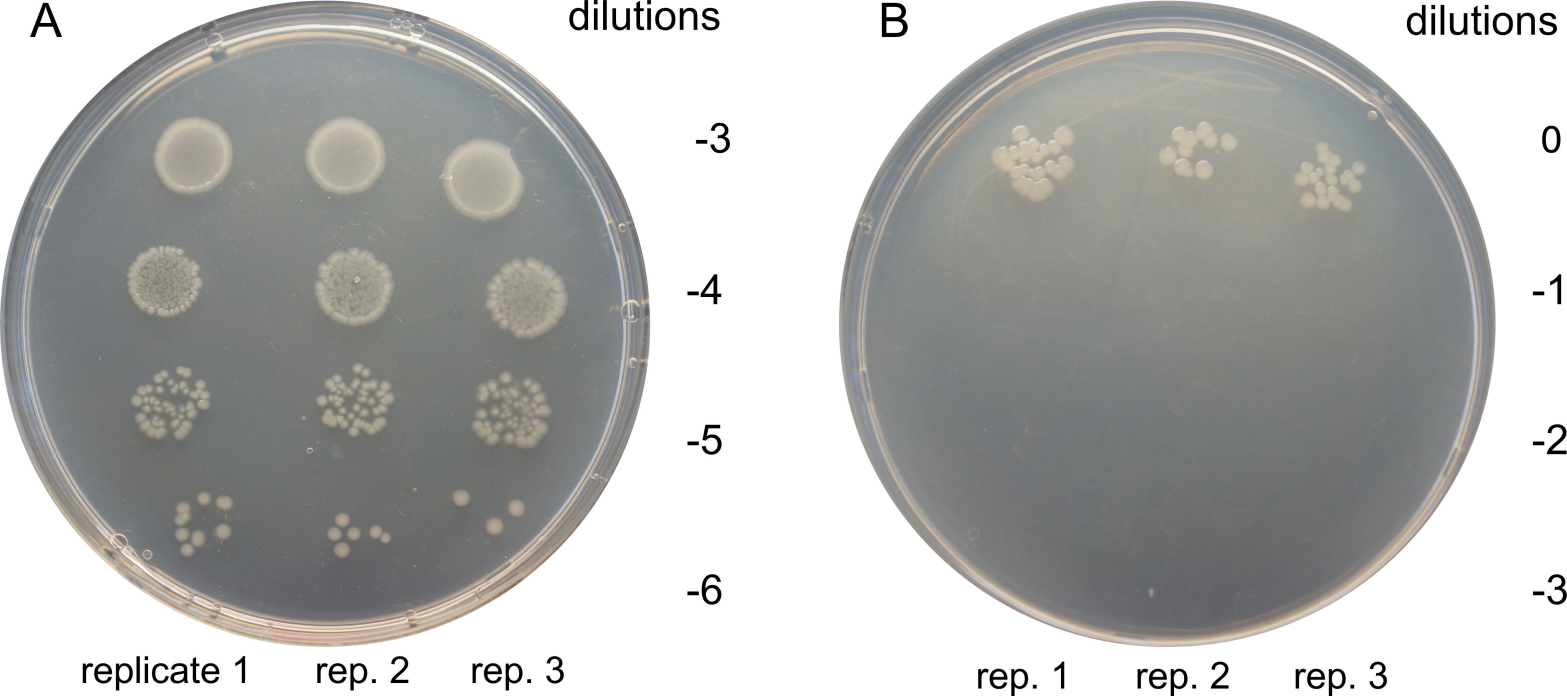
Colony counts on T3 agar plates (**A**) before and (**B**) after heat treatment at 80°C.

To assess heat resistance, cultures were subjected to 80°C for 30 min. Before heating, plating from the 10⁻⁵ dilution yielded colony counts of 45, 47, and 48 across replicates (Fig. 4A), corresponding to a CFU/mL of (4.67 ± 0.08) × 10⁸. After heating, plating of undiluted culture yielded colony counts of 22, 12, and 21 (Fig. 4B), respectively, resulting in a CFU/mL of (1.83 ± 0.32) × 10³. Notably, viable cells were recovered after heat treatment at 80°C, despite the absence of morphologically detectable endospores.

Gram staining showed a Gram-negative reaction (Fig. 2C). The string test revealed the formation of long, viscous filaments extending from the inoculating loop within the first 30 seconds, supporting the Gram-negative phenotype of the isolate.

**Fig 5:**
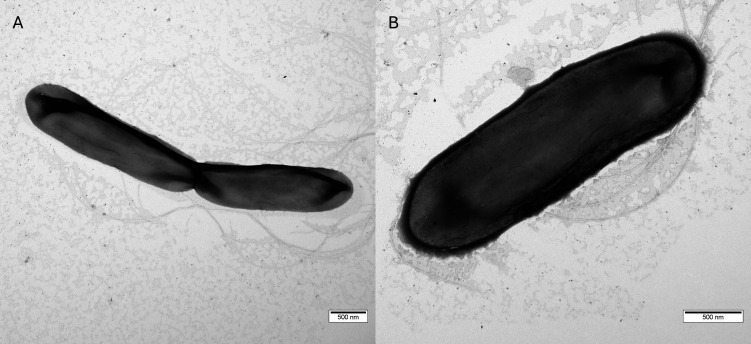
Transmission electron micrographs of the nc5.1 strain. (**A**) Vegetative cell in division. (**B**) Single vegetative cell. Peritrichously arranged filaments are visible on the cell surface. Scale bar = 500 nm.

Transmission electron microscopy further revealed rod-shaped vegetative cells ranging from 2.2 to 3.3 µm in length and 0.5 to 0.7 µm in width (Fig. 5). Cells were surrounded by peritrichously arranged filaments with diameters of 11 ± 2 nm and lengths of up to 6.6 µm. These ultrastructural observations are consistent with the motile phenotype of strain nc5.1.

Motility was confirmed, with individual cells displaying active, directed movement across the entire field of view. These observations were complemented by transmission electron microscopy, which provided further details on cell morphology.

### Sequencing and annotation of the nc5.1^T^ strain

To disentangle the taxonomic attribution of the nc5.1 strain, we first assembled and annotated its genome. The genome assembly consisted of two circular contigs with a total length of 3,954,588 bp (3,913,821 and 40,767 bp, respectively). The GC content reached 35.61 mol%. The assembly was characterized by high coverage depth (435) and estimated completeness (100%), coupled with low contamination (1.99%) and almost full representation (99.1%) of single-copy BUSCO (20) markers from the bacillales_odb10 database. The annotated genome included 3,946 genes, 3,837 of which were CDS. The properties of the genome suggest high quality and a full level appropriate for performing downstream large-scale taxonomic analysis.

### The comparative analysis of the nc5.1^T^ strain reveals that it represents a novel species of the *Psychrobacillus* genus

**Fig 6:**
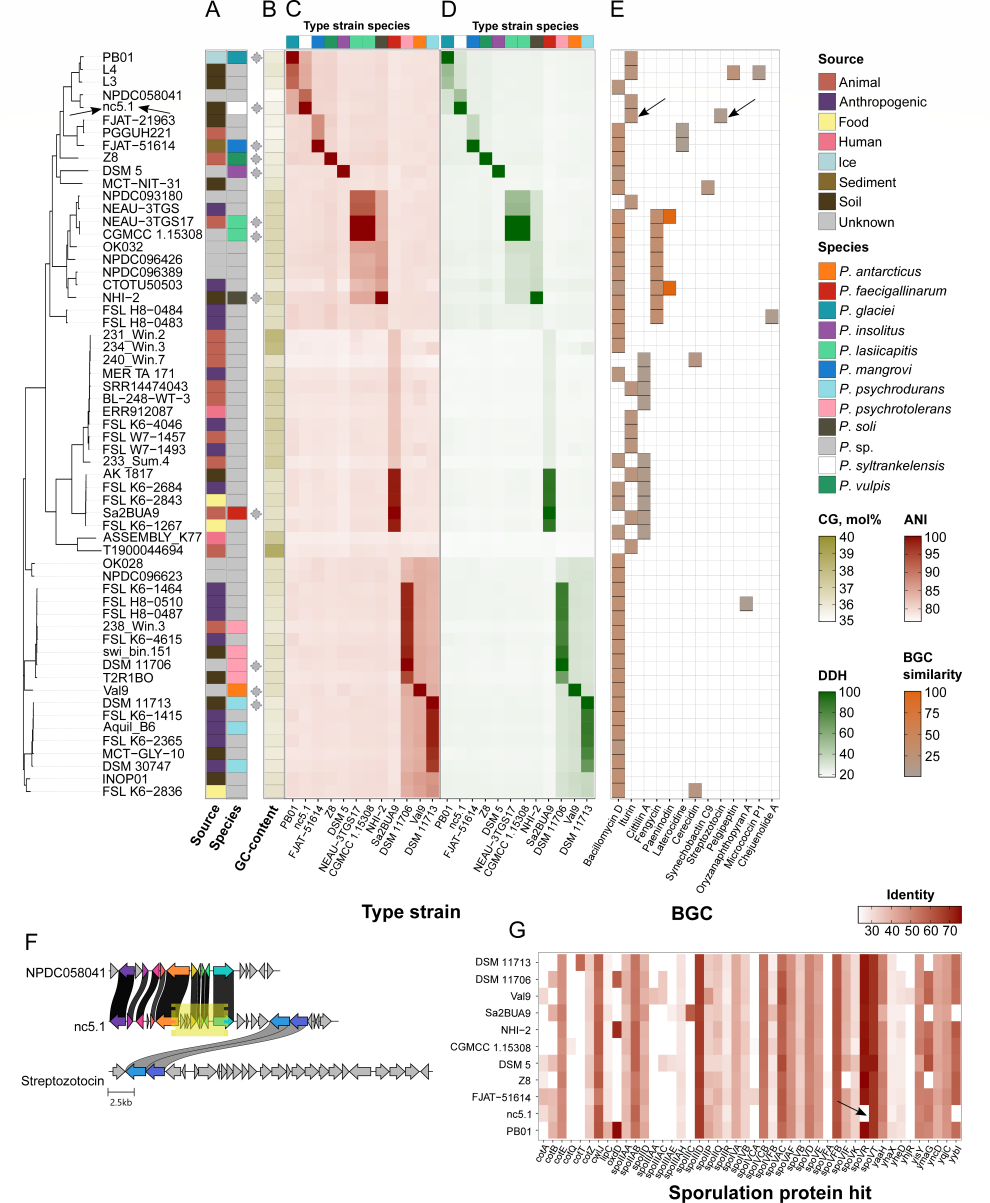
Key taxonomic and genomically-derived metabolic characteristics of the *P. syltrankelensis* strain nc5.1^T^ in the context of all genomes of the *Psychrobacillus* genus. All the plots are ordered according to the leftmost maximum likelihood (ML) phylogeny reconstructed on the core genome. The black arrow highlights the strain nc5.1^T^ and its properties. (**A**) General metadata of strains included in the analysis, namely, the isolation source and the species attributions. The color of the tiles corresponds to the respective metadata. The gray diamond figures mark type strains of distinct species. (**B**) The GC content (mol%) distribution in the *Psychrobacillus* genus representatives. The intensity of the color is proportional to the percentage. (**C**) Pairwise whole genome comparisons of the analyzed assemblies with the reference genomes of type strains of distinct *Psychrobacillus* species using the average nucleotide identity (ANI) and (**D**) digital DNA-DNA hybridization (DDH). The adjacent stripes on top of the similarity heatmaps indicate the species of the type strain in comparison. In both figures (**C and D**), the colored strip at the top of the heatmaps indicates to which species the type strains designated at the bottom of the figure belong. (**E**) The presence patterns of known biosynthetic gene clusters (BGCs) found among *Psychrobacillus* species. The color reflects the average core genes' similarity with the closest known BGC. (**F**) Composition of suspected streptozotocin-producing homolog found in the nc5.1^T^ genome. The closest cluster from the data set, coupled with the reference BGC, is presented. The relative position of the putative HGT event is pictured as the yellow rectangle. (**G**) Homologs of known sporulation regulators identified in the analyzed genomes. The color is proportional to the identity estimates, and the black arrow points at the factors absent in the nc5.1^T^ genome.

Having obtained the full high-quality genome of the strain nc5.1^T^, we moved on to identify its taxonomic origin. We first used the average nucleotide identity (ANI)-based approach to retrieve the closest genome deposited in the NCBI RefSeq (10) resource. The closest strain was *Psychrobacillus* sp. NPDC058041 (91.55%) with unknown species attribution, while the closest known species corresponded to *Psychrobacillus glaciei* PB01^T^ (84.31%). Since these parameters indicate that our strain is a novel species, we collected all genomes (59 with our strain included) belonging to the genus of *Psychrobacillus* available in the NCBI Assembly resource and reconstructed the core phylogeny accordingly (Fig. 6).

Notably, of the strains with known metadata (Fig. 6A), the majority represented those isolated from anthropogenic (16 isolates) and animal (13) sources. Soil-borne strains, the same as our strain, corresponded to 12 genomes in total. We have not found any evident relationships between the isolation source and the phylogenetic group. The vast majority of the data set (42 strains) lacked species annotations (Fig. 6A), while for the classified ones, *P. psychrotolerans* (4) and *P. psychrodurans* (3) prevailed. A total of 10 distinct known species were presented. The mean GC content of the genomes was 36.7 mol%, which is close to our strain (35.61 mol%). The values ranged from 35.3 to 39.2 mol%; however, the genomes with higher GC content did not form a distinct clade and were sporadic (Fig. 6B), assuming the quality determines GC content, while, in general, it is relatively uniform among the genus.

To explicitly classify our strains taxonomically, we analyzed the well-established methods relative to the type strains of the known species. As mentioned above, the ANI estimates showed *P. glaciei* PB01^T^ to be closest to our strain (Fig. 6C), which corroborated the presence of the phylogenetic clade encompassing the *P. glaciei* reference genome. Apart from the 11-leaf phylogenetic clade without isolate (Fig. 6C) with ANI estimates spanning from 91.55% to 80.26%, for the remaining strains, the ANI values relative to the strain nc5.1^T^ did not exceed 80% of similarity. The digital DNA-DNA hybridization (DDH) characteristics resembled those related to ANI, with a starker contrast obtained (Fig. 6D). The DDH with *Psychrobacillus* sp. NPDC058041 was 49.6%, while for the type strain *P. glaciei* PB01^T^, it reached 31% only. On average, the DDH similarity between the nc5.1^T^ and the genomes in the data set was 22.8%. Despite such a conspicuous whole-genome difference, the average pair-wise difference in 16S rRNA sequences, another prominent taxonomic marker, hovered around 97.8%, and the mean similarity relative to our strain reached 98.9%. It was also high when comparing our isolate with the closest type strains, namely, *P. glaciei* PB01^T^ (99.6%), *P. vulpis* Z8^T^ (99.7%), and *P. mangrovi* FJAT-51614^T^ (99.1%). To prove the correctness of 16S rRNA-based taxonomic assignment, we applied the Sanger sequencing technique using two pairs of primers and obtained a high-confidence overlapping region encompassing 1,090 bp out of 1,553. Similarly, the identity with *P. glaciei* PB01^T^ reached 99.6%, thus corroborating the genome-based inference. Interestingly, the rML phylogeny reconstructed in 16S rRNA sequences showed only 61.6% topological identity with the reference core SNPs-based phylogeny.

In addition to general genomic comparisons, we dug for functional characteristics of the strains from their genomic data in terms of metabolic capacities maintained by secondary metabolites associated with biosynthetic gene clusters (BGCs; Fig. 6E). On the whole, the most widespread BGCs were operons responsible for the synthesis of bacillomycin D (38), iturin (12), cittilin A (10), and fengycin (8). Other clusters were doubletons (3) or singletons (6). The strain nc5.1^T^ contained two clusters (Fig. 6E) producing iturin (22.2%) and streptozotocin (7.7%). The latter represents a remote putative homolog of the known cluster due to extremely low similarity. The closest gene cluster relative to the studied strain was detected in the NPDC058041 strain, and 10 genes with the same genomic topology were reported (Fig. 6F). None of the isolates, except the nc5.1^T^ genome, possessed orthologous regions to the reference streptozotocin cluster. Notably, we found a signal of HGT using Alien_hunter v1.7 (21) associated with the respective BGC region, which implies a possible HGT event. The putative HGT-related signal lies upstream of the two homologs constituting the reference BGC (Fig. 6F).

Finally, we inspected the genomic landscape associated with the sporulation process to explain the distinct behavior of the nc5.1^T^ strain. For such purposes, we searched for the components controlling sporulation using the in-house data set of known sporulation regulation proteins. From a total of 80 well-studied sequences from the SubtiWiki database (22), 393 hits with the whole genomic data set were reported (Fig. 6G). Generally, *Psychrobacillus* sp. type strains contained from 33 to 28 homologs, and nc5.1^T^ harbored 34 homologs, while 29 of them were found in all genomes. It is noteworthy that the *spoVR* and *yybI* genes were absent in the nc5.1^T^ genome. The former was identified in all remaining renomes, while the latter was absent in the Sa2BUA9^T^ strain as well.

All things considered, the genomic criteria for species delineation, i.e., ANI and DDH (Table 1), implied that the strain nc5.1^T^ should be considered a novel, distinct species, which we termed *P. syltrankelensis*. The strain possesses a unique BGC composition and a set of morphological and genomic differences with known representatives of the genus.

**TABLE 1:** Comparisons of general genomic features between the analyzed strain nc5.1^T^ and the closest type strains of the *Psychrobacillus* genus^*a*^

Feature/strain	nc5.1^T^	PB01^T^	Z8^T^	FJAT-51614^T^	NHI-2^T^	DSM 5^T^
Species	*P*. *syltrankelensis*	*P*. *glaciei*	*P*. *vulpis*	*P*. *mangrovi*	*P*. *soli*	*P*. *insolitus*
Genome length, bp	3,931,821	4,351,338	4,023,262	4,202,498	4,224,866	3,288,307
Number of genes	3,946	4,243	3,995	4,146	4,163	3,350
GC content, mol%	35.61	35.97	35.9	35.73	37.12	36.02
ANI	-	84.31	80.33	80.34	79.82	79.23
DDH	-	31	24.5	24.5	22.8	22.7
16S rRNA similarity	-	99.61	99.66	99.1	99.44	99.16

^*a*^ The “-” symbol indicates self-comparisons.

### Physiological, biochemical, and chemotaxonomic characteristics

Since genomic analyses indicated that *P. syltrankelensis* nc5.1^T^ represents a novel species, additional phenotypic characterization was carried out, including catalase and urease tests, substrate utilization profiling, and cellular fatty acid analysis.

The strain was catalase-positive and urease-negative. The Biolog GEN III MicroPlate assay showed that *P. syltrankelensis* nc5.1^T^ was able to assimilate 46 different carbon sources, covering a broad spectrum of carbohydrates, alcohols, amino acids, organic acids, and other compounds (Table 2). It did not utilize D-maltose, D-trehalose, D-cellobiose, gentiobiose, sucrose, D-turanose, stachyose, D-raffinose, α-D-glucose, glycerol, D-fructose-6-PO_4_, D-serine, L-alanine, pectin, p-hydroxy- phenylacetic acid, methyl pyruvate, citric acid, bromo-succinic acid, γ-amino-butyric acid, α-hydroxy- butyric acid, β-hydroxy-D,L- butyric acid, α-keto-butyric acid, acetoacetic acid, propionic acid, and formic acid. The strain also showed tolerance to aztreonam, nalidixic acid, and potassium tellurite.

**TABLE 2:** Carbon sources assimilated by *P. syltrankelensis* nc5.1^T^ according to the biolog GEN III MicroPlate assay

Carbohydrates & derivatives	Alcohols	Amino acids	Organic acids & derivatives	Other compounds
Dextrin α-D-Lactose D-Melibiose β-Methyl-D-glucoside D-Salicin N-Acetyl-D-glucosamine N-Acetyl-β-D-mannosamine N-Acetyl-D-galactosamine D-Glucose-6-PO_4_ D-Mannose D-Fructose D-Galactose 3-Methyl glucose D-Fucose L-Fucose L-Rhamnose	D-Sorbitol D-Mannitol D-Arabitol myo-inositol	D-Aspartic acid L-Arginine L-Aspartic acid L-Glutamic acid L-Histidine L-Serine L-Pyroglutamic acid	N-Acetylneuraminic acid L-Galactonic acid lactone D-Gluconic acid D-Glucuronic acid D-Galacturonic acid Glucuronamide Mucic acid Quinic acid D-Saccharic acid D-Lactic acid methyl ester L-Lactic acid α-Keto-glutaric acid D-Malic acid L-Malic acid Acetic acid	Inosine Gelatin Glycyl-L-proline Tween 40

Comparison of carbon source assimilation among *P. syltrankelensis* nc5.1^T^ and the type strains of *P. glacei* PB01^T^ and *P. vulpis* Z8^T^ revealed several distinctive features (Table 3). *P. syltrankelensis* nc5.1^T^ assimilated D-mannitol, D-mannose, D-sorbitol, and inositol, but did not utilize D-maltose, sucrose, or D-raffinose. In contrast, *P. glacei* PB01^T^ was positive for maltose but negative (or weakly positive) for the other substrates, whereas *P. vulpis* Z8^T^ did not assimilate any of the tested carbon sources.

**TABLE 3:** Differential carbon source utilization by *P. syltrankelensis* nc5.1^T^ and genomically close type strains^*a*^

Carbon source	*P*. *syltrankelensis* nc5.1^T^	*P. glaciei* PB01^T^	*Psychrobacillus vulpis* Z8^T^
D-Mannitol	**+**	w	**–**
D-Maltose	**–**	**+**	**–**
D-Mannose	**+**	ND	**–**
D-Sorbitol	**+**	**–**	ND
Sucrose	**–**	**–**	ND
Inositol	**+**	**+**	ND
D-Raffinose	**–**	**–**	ND

^*a*^ +, Positive; −, negative; w, weak; ND, no data available. Data for *P. glacei* PB01T were taken from Choi et al. (4), and data for *P. vulpis* Z8T from Rodríguez et al. (5).

The major cellular fatty acids of *P. syltrankelensis* nc5.1^T^ were C_14:1_ (52.8%), C_14:0_ (16.0%) and C_16:0_ (7.7%), with moderate amounts of C_15:0_ (3.7%), C_18:1_ (3.6%), and C_18:0_ (2.2%) (Table 4). In comparison, the available data for *P. glaciei* PB01ᵀ indicate only a trace amount of C_14:0_ (0.4%), coupled with a similar proportion of C_16:0_ (7.6%) and a higher content of C_18:0_ (9.1%) (4). Comparable data on the fatty acid profile of *P. vulpis* Z8ᵀ have not yet been published.

**TABLE 4:** Cellular fatty acid composition of *P. syltrankelensis* nc5.1^T^^*a*^

Cellular fatty acid	*P*. *syltrankelensis* nc5.1^T^	*P. glaciei* PB01ᵀ (4)
C_11:0_	0.16	ND
C_12:0_	0.22	ND
C_13:0_	3.33	ND
C1_4:0_	16.04	0.4
C_14:1_	52.80	ND
C_15:0_	3.68	ND
C_15:1_	0.87	ND
C_16:0_	7.73	7.6
C_16:1_	1.10	ND
C_16:2_	7.17	ND
C_16:3_	0.00	ND
C_18:0_	2.21	9.1
C_18:1_	3.61	ND
C_18:2_	0.92	ND
C_18:3_	0.00	ND
C_20:1_	0.00	ND
C_21:0_	0.15	ND

^*a*^ Values are expressed as percentages of the total fatty acids. ND, no data available.

These phenotypic differences support the delineation of *P. syltrankelensis* nc5.1^T^ as a novel species within the genus *Psychrobacillus*.

### Impact on the viability of PANC-1 cells

**Fig 7:**
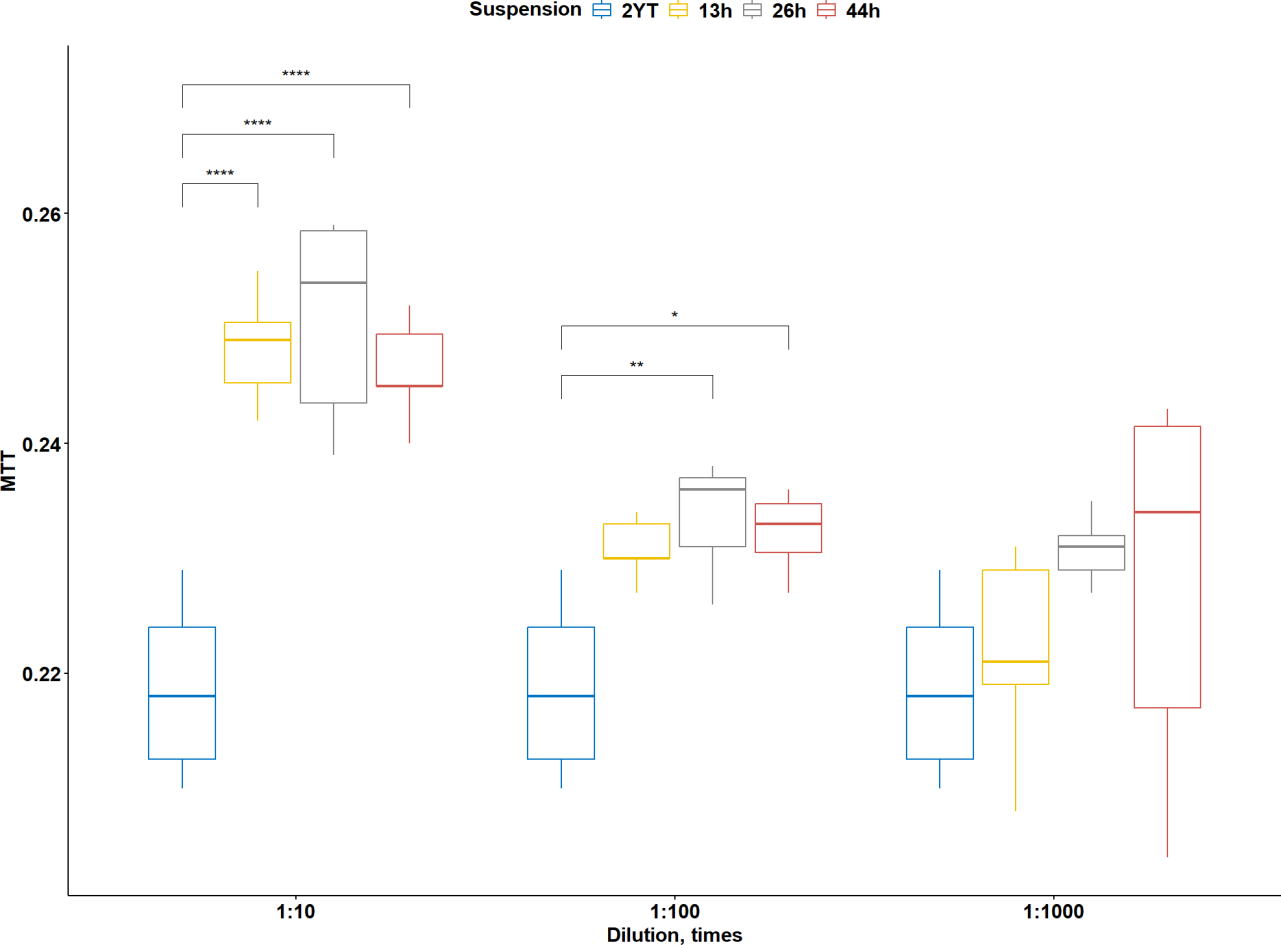
Effect of *P. syltrankelensis* nc5.1ᵀ supernatants on PANC-1 cell viability after 24-h exposure. Supernatants from 13, 26, and 44 h cultures were applied at 1:10, 1:100, and 1:1,000 dilutions. Viability values were normalized to the 2YT medium control. Data are presented as box plots, and whiskers indicate the data range. Statistical significance relative to the 2YT control is indicated above the plots. * *P* < 0.05, ** *P* < 0.01, **** *P* < 0.0001*.*

Since the genomic analysis of *P. syltrankelensis* nc5.1^T^ revealed biosynthetic gene clusters with possible inhibitory effects on cell growth, MTT assays were conducted to evaluate the impact of culture supernatants on the human pancreatic carcinoma cell line PANC-1. Supernatants were collected after 13, 26, and 44 h of cultivation and applied at 1:10, 1:100, and 1:1,000 dilutions. The 2YT medium control was used as the reference group for all comparisons. Parallel cell-free control wells containing complete medium supplemented with the respective supernatant dilutions did not produce a measurable MTT signal, indicating the absence of direct chemical reduction of the tetrazolium dye by bacterial metabolites. No inhibitory effects were observed under any tested condition. Instead, an increase in MTT signal was detected in a concentration-dependent manner (Fig. 7).

At a 1:10 dilution, supernatants from all three time points (13, 26, and 44 h) significantly increased the MTT signal relative to 2YT control, by approximately 14.2%, 16.5%, and 12.4%, respectively, with no differences between time points. At 1:100, significant increases were observed at 26 h (~8.3%) and 44 h (~6.9%), while the 13 h sample did not differ from 2YT. At 1:1,000, none of the time points showed a statistically meaningful effect.

Overall, the effect of *P. syltrankelensis* nc5.1ᵀ supernatants on PANC-1 cells was moderate and concentration-dependent, without significant differences between growth stages.

## DISCUSSION

In this study, we isolated and comprehensively characterized strain *P. syltrankelensis* nc5.1ᵀ, inhabiting mountain soil near Lake Syltran-Kel. Having employed both morphological genome-wise and functional assays, we revealed a combination of unusual traits that together support the distinction of this isolate as a novel species within *Psychrobacillus*.

The strain *P. syltrankelensis* nc5.1ᵀ exhibited a Gram-negative staining response, as determined by conventional Gram staining and confirmed by the 3% KOH string test, differentiating it from its predominantly Gram-positive counterparts within the family of *Bacillaceae* (23). Nonetheless, similar exceptions have been documented across related taxa. For example, *Bacillus horti* was described as a Gram-negative alkaliphilic species within *Bacillus* (24). Likewise, *Paenibacillus mobilis* was characterized as a Gram-negative yet endospore-forming species (25). Within *Lysinibacillus*, both *L. timonensis* and *L. composti* have been reported as Gram-negative, spore-forming bacteria (26, 27). These examples indicate that Gram-negative staining can indeed occur within *Bacillaceae* species.

The absence of visible spores in nc5.1ᵀ under our laboratory conditions also warrants consideration. Sporulation in *Bacillaceae* is tightly regulated and typically triggered by specific stressors (e.g., nutrient limitation, desiccation) (28); therefore, standard rich media might not induce this developmental pathway. However, even under sporulation-inducing conditions applied in this study, no morphologically detectable endospores were observed, suggesting that the asporogenous phenotype cannot be explained solely by the cultivation settings. In addition, experimental-evolution studies demonstrate that *Bacillus subtilis* populations can accumulate mutations that reduce or abolish sporulation capacity, underscoring that asporogenous phenotypes can arise and persist under certain selection conditions (29). Comparative genomics further shows lineage-specific erosion of sporulation gene sets across *Firmicutes*, consistent with multiple, independent routes toward diminished sporulation potential (30). Genome mining of the *Psychrobacillus* genus representatives revealed that our strain lacked genes coding for SpoVR and YybI, representing a regulatory component responsible for the formation of the spore cortex (31) and the major inner coat protein (32), respectively. Therefore, the genetic composition of the strain could explain the unexpected behavior of the strain and dependence on specific conditions irreproducible by standard techniques. Notably, although no morphologically detectable endospores were observed, a minor but reproducible viable fraction persisted after exposure to 80°C. This observation does not necessarily contradict the asporogenous phenotype. In light of the genomic features discussed above, including the apparent erosion of specific sporulation-associated genes, it is conceivable that the sporulation cascade may be initiated but not completed. Such incomplete activation could generate physiologically stress-tolerant states without culminating in fully mature, morphologically recognizable endospores, thereby conferring transient thermal resistance. Given the atypical morphological features, we next applied genome-based comparisons to resolve the taxonomic position of the strain.

Current bacterial species delineation criteria rely on genomic estimates, primarily ANI and DDH, with the thresholds of 95% and 70%, respectively (19, 33). The observed values of the isolate nc5.1^T^ termed *P. syltrankelensis* fell below these thresholds relative to the closest type strain *P. glaciei* PB01^T^ (Table 1), confirming its status as a novel species. Consistent with previous reports on the genus, 16S rRNA sequence similarity was insufficient for species demarcation (1, 4, 5, 7), favoring genome-wide comparisons. Moreover, the general distribution of ANI and *in silico* DDH estimates with the *Psychrobacillus* genus type strains suggests uncharacterized taxonomic diversity (Fig. 6C and D).

In addition to genome characterization, we inspected chemotaxonomic markers. Fatty acid composition is a well-established parameter in *Bacillaceae* taxonomy (34). The fatty acid profile of *P. syltrankelensis* nc5.1ᵀ was dominated by mid-chain straight-chain fatty acids, notably monounsaturated C_14:1_ (52.8%), saturated C_14:0_ (16.0%), and C_16:0_ (7.7%). Such a pattern is unusual among *Bacillaceae*, where branched-chain fatty acids often prevail (34). Elevated levels of straight-chain unsaturated fatty acids are generally associated with increased membrane fluidity (35), and modulation of fatty-acid saturation is a well-established mechanism of cold adaptation in psychrotolerant and psychrophilic bacteria (36). However, in *P. syltrankelensis* nc5.1ᵀ, this trait did not enable psychrotolerance, since the strain failed to grow below +20°C in laboratory tests. Instead, the high proportion of unsaturated C_14:1_ could represent a species-specific strategy to modulate membrane fluidity and stability under moderate but fluctuating conditions, rather than an adaptation to growth at near-freezing temperatures *per se*. Differences in the relative abundances of major fatty acids compared with *P. glaciei* further support the recognition of nc5.1ᵀ as a distinct species.

The metabolic profile of *P. syltrankelensis* nc5.1ᵀ is a stable phenotypic marker, differentiating it from closely related taxa, such as *P. glaciei* PB01ᵀ and *P. vulpis* Z8ᵀ, via distinct patterns of substrate use and corresponding to species-specific ecological adaptations (37). Genome mining revealed a mutually exclusive distribution of BGCs for cyclic lipopeptides. The strains possessed either bacillomycin D or iturin clusters (Fig. 6E). Our strain *P. syltrankelensis* nc5.1^T^ belonged to the iturin-producing group, implying possible antifungal properties shown for this compound (38–40). Additionally, nc5.1^T^ contained a divergent BGC (7.7% similarity) associated with streptozotocin biosynthesis. Streptozotocin is known for cytotoxicity, especially toward the pancreatic cells (41, 42). The moiety is synthesized by the soil bacterium *Streptomyces achromogenes* (42). Given the low similarity and gene composition (Fig. 6F), this locus likely represents a common origin of specific enzymatic genes rather than a direct cluster transfer from *Streptomyces,* potentially encoding a novel bioactive compound.

However, functional validation yielded intriguing results compared with genomic predictions. Despite harboring BGCs responsible for the synthesis of bioactive compounds, the culture supernatants of the strain produced an unexpected outcome in the human pancreatic carcinoma cell line PANC-1. Culture supernatants of *P. syltrankelensis* nc5.1ᵀ moderately stimulated the viability of PANC-1 cells in MTT assays rather than inhibiting cell growth, despite the presence of BGCs for iturin and a streptozotocin-like metabolite in its genome. The stimulatory rather than inhibitory effect on PANC-1 cells may reflect a hormetic effect, i.e., the induction of cellular metabolism by low concentrations of stress-inducing compounds (43). Alternatively, bacterial supernatants could contain nutritional or signaling molecules, such as peptides or organic acids, that act as growth enhancers under *in vitro* conditions (44). Since tetrazolium-based assays may be affected by redox-active compounds (45), the possibility of direct chemical reduction of MTT by bacterial metabolites was specifically addressed. Parallel cell-free controls containing supernatant dilutions did not produce a detectable signal, indicating that the observed increase required the presence of viable cells and was not attributable to assay interference. Taken together, the results highlight the value of integrating genomic and functional approaches to uncover unexpected phenotypes in secondary metabolite-producing bacteria.

### Conclusion

The integrative characterization of strain *P. syltrankelensis* nc5.1ᵀ revealed a unique combination of traits that expand our understanding of diversity within the family of *Bacillaceae*. Despite its belonging to the *Psychrobacillus* genus, the isolate displayed atypical Gram-negative staining and an absence of sporulation under laboratory conditions, underscoring the limitations of typical morphological criteria. Whole-genome comparisons unambiguously distinguished nc5.1ᵀ from its closest relative, *P. glaciei*, and chemotaxonomic markers, such as the unusual dominance of straight-chain fatty acids, further reinforced its status as a distinct species. Physiological assays further highlighted a distinct metabolic profile, marked by broad assimilation of simple sugars and amino acids combined with the inability to metabolize disaccharides and organic acids, suggesting ecological specialization in nutrient-limited soils. Genome mining revealed biosynthetic gene clusters producing iturin-like lipopeptides and a highly divergent locus resembling streptozotocin-producing BGC, thus highlighting the unexplored potential for natural product discovery. Unexpectedly, culture supernatants moderately stimulated the viability of human pancreatic cells in MTT assays, illustrating the complexity of genotype-to-phenotype translation in secondary metabolite producers. Taken together, these findings support that the nc5.1ᵀ isolate represents a type strain of a novel species, *Psychrobacillus syltrankelensis* sp. nov., and emphasize the value of combining genomic, ecological, and functional description to uncover hidden diversity and biotechnological potential within *Bacillaceae*.

## MATERIALS AND METHODS

### Sample collection and storage

**Fig 8:**
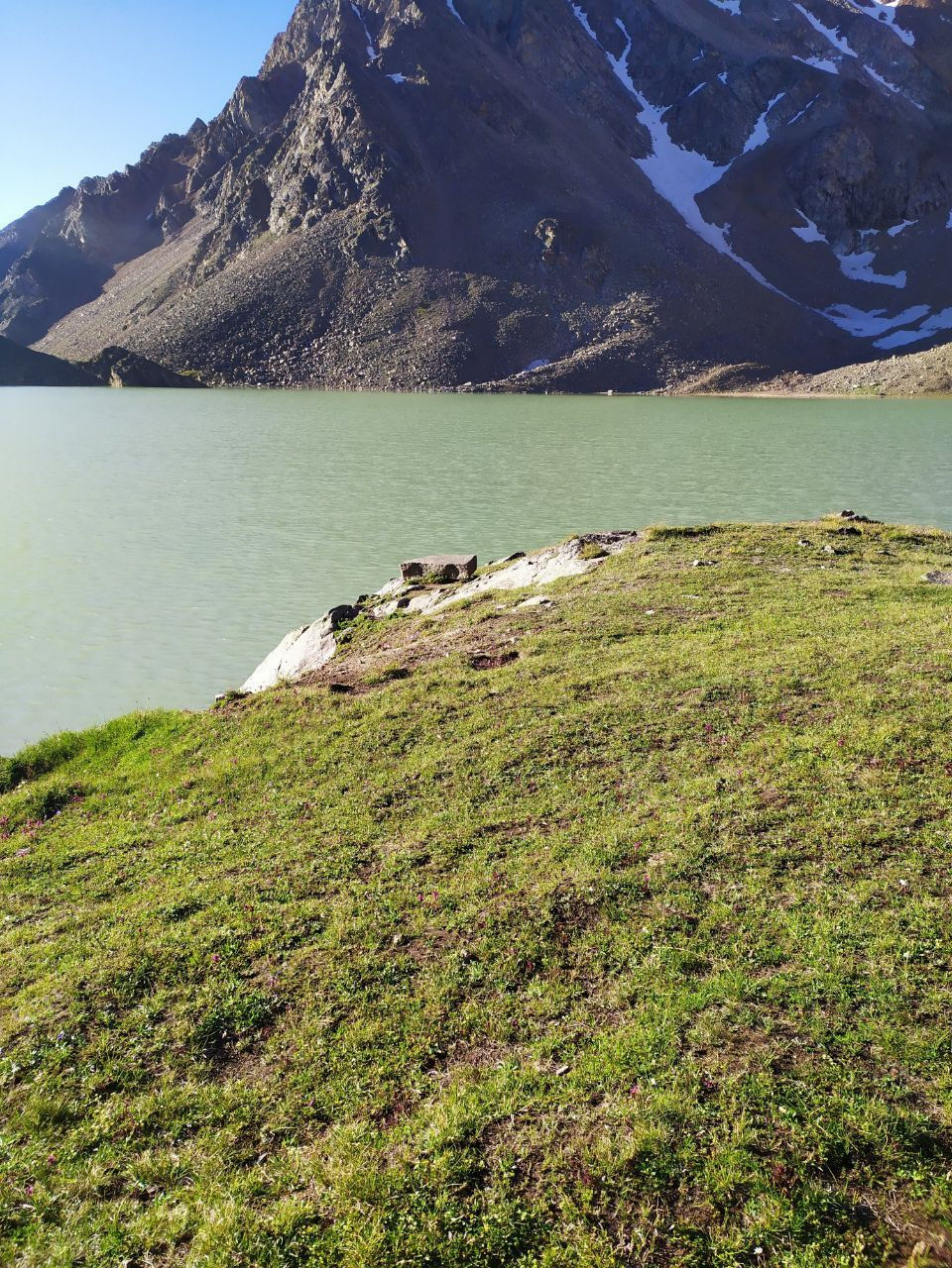
Soil sampling location.

Soil material was collected in July 2023 from five subsites within an area of approximately 1 × 1 m at a depth of 3–5 cm near Lake Syltran-Kel, Kabardino-Balkarian Republic, Russia (43.327,194 N, 42.673,167 E) (Fig. 8), following a previously described sampling protocol (46). The sample was subsequently transported to the laboratory and stored at 4°C–10°C until further processing.

For the isolation of spore-forming bacteria, about 0.2 g of soil was suspended in 2 mL of sterile water and thoroughly mixed for 10 min. The suspension was divided into 1-mL aliquots, transferred into microcentrifuge tubes, and subjected to heat treatment at 80°C for 30 min to eliminate vegetative cells and non-spore-forming organisms. Serial tenfold dilutions (10⁻¹ to 10⁻³) were prepared, and 200 μL of each dilution was plated onto T3 agar, a medium favorable for sporulation (per liter: tryptone, 3 g; tryptose, 2 g; yeast extract, 1.5 g; NaH₂PO₄·H₂O, 6.9 g; MnCl₂·4H₂O, 0.008 g; agar, 15 g; pH 6.8) (18). Plates were incubated at 28°C for 72 h. Colonies with characteristic morphology were selected and purified by repeated transfers on fresh T3 agar until pure cultures were obtained.

### Determination of growth parameters

Growth was evaluated under varying pH, NaCl concentrations, and temperatures as described by (47, 47) with minor modifications. Cultures were grown in 2YT broth (per liter: tryptone, 16 g; yeast extract, 10 g; NaCl, 5 g) (48). pH tolerance was examined in medium adjusted to values between 4.0 and 11.0. NaCl tolerance was tested in medium supplemented with 0–15% (w/v) NaCl. Optical density at 600 nm (OD₆₀₀) was recorded at 30-min intervals for 48 h using a CLARIOstar Plus plate reader (BMG LABTECH, Ortenberg, Germany), and growth curves were generated. For temperature profiling, cells precultured on 2YT agar were resuspended in liquid medium to an OD₆₀₀ of 0.2, diluted to 0.002, and incubated in an orbital shaker for 24 h at temperatures between 5°C and 45°C. Samples were taken at 4, 8, and 24 h, and OD₆₀₀ was measured with an IMPLEN DiluPhotometer (Implen GmbH, Munich, Germany). The final 24-h OD values were used to construct the temperature growth profile.

### Morphological characterization of the strain

Standard colony features (shape, elevation, optical properties, color, surface structure, and margin), as well as vegetative cell morphology, were assessed after cultivation on 2YT agar (per liter: tryptone, 16 g; yeast extract, 10 g; NaCl, 5 g) (48) at 30°C. Colonies and cells were evaluated after 24 h. For light microscopy, ~10 µL of culture was taken with a microbiological needle, suspended in sterile deionized water, air-dried, stained for 10–15 min with Coomassie Brilliant Blue (Bio-Rad Laboratories, Hercules, CA, USA), rinsed with distilled water, and observed at ×1,000 magnification for morphological examination using a Carl Zeiss Axio Imager two microscope (Jena, Germany).

Endospore formation was assessed using the Schaeffer–Fulton staining method (49). After 120 h of cultivation on T3 agar medium (18) at 28°C and after 120 h of cultivation in Schaeffer’s liquid sporulation medium at 28°C, smear preparations were made on microscope slides. The fixed smear was covered with 5% aqueous malachite green (LenReaktiv, Saint Petersburg, Russia) and heated over a flame until steam appeared; this step was repeated four times. After rinsing with water for 30 s, the smear was air-dried and counterstained with 0.5% aqueous safranin T (LenReaktiv, Saint Petersburg, Russia) for 30 s. Following a final rinse with water, preparations were examined microscopically.

To further evaluate sporulation capacity, the isolate was incubated for 120 h at 28°C with agitation at 190 rpm in a Series 44 Innova incubator shaker (New Brunswick Scientific, USA) using a 750-mL Erlenmeyer flask containing 40 mL of Schaeffer’s medium (per liter of distilled water: 0.1% KCl, 0.01% MgCl₂, 1.0 mM Ca(NO₃)₂, 0.01 mM MnCl₂, 0.001 mM FeSO₄, and 8 g nutrient broth) (50). After incubation, smears were prepared and stained, as described above.

To assess resistance to heat treatment, a 12 h liquid culture grown in T3 medium (18) at 28°C with agitation (190 rpm) was divided into two 1,000-µL aliquots. One aliquot was subjected to heat treatment at 80°C for 30 min in a water bath (BioSan WB-4MS, Latvia), while the second aliquot was kept at room temperature (22°C ± 2°C). The experiment was performed in three biological replicates. After treatment, serial dilutions were prepared (10⁻³–10⁻⁶ for untreated samples and 10⁰–10⁻³ for heat-treated samples), and 10 µL of each dilution was plated onto T3 agar. Plates were incubated at 28 °C for 18 h. Colony-forming units (CFU/mL) were calculated based on colony counts and expressed as mean ± SEM.

Ultrastructural observations of vegetative cells were performed with a Jeol JEM-1400 transmission electron microscope (JEOL Corp., Tokyo, Japan) equipped with a Veleta CCD camera (Olympus-SIS, Münster, Germany). Cell suspensions were deposited onto copper grids coated with formvar–carbon films (Electron Microscopy Sciences, Hatfield, PA, USA) and negatively stained with 1% aqueous uranyl acetate. TEM analyses were conducted on cultures grown for 24 h in liquid 2YT broth (48).

Gram staining was performed using a commercial staining kit (LenReaktiv, Saint Petersburg, Russia) according to the manufacturer’s instructions. Briefly, fixed smears of 24-h cultures were sequentially stained with crystal violet, treated with Lugol’s iodine solution, decolorized with ethanol, and counterstained with fuchsin, followed by light microscopy at ×1,000 magnification. To confirm Gram staining results, a string test using 3% potassium hydroxide (KOH) was performed (51). A drop of 3% KOH solution was placed on a clean microscope slide. A full 1-µL loop of a 24-h culture grown on T3 agar medium was emulsified in the drop and vigorously mixed with the loop for 60 s. The loop was then carefully withdrawn from the drop, and the result was assessed.

Motility was assessed using a crushed-drop preparation under oil immersion (×1,000) with a Zeiss Axio Imager 2 microscope (Jena, Germany). The strain was considered motile when a directed movement was observed, even if it was detected in only a small proportion of cells (52). Cells showing no movement or only uniform displacement due to convection currents were classified as non-motile (53).

### 16S rRNA gene sequencing

DNA extraction, PCR amplification, and purification of PCR products were performed, as described previously (54) with minor modifications. The 16S rRNA gene was amplified using two common primer sets, 27F (5′-AGAGTTTGATCCTGGCTCAG-3′) with 1492R (5-ACGGYTACCTTGTTACGACTT-3′) (55) and 16S RNA Forward (5′-AGAGTTTGATCCTGGCTCAG-3′) with 16S RNA Reverse (5′-CTTGTGCGGGCCCCCGTCAATTC-3′ (Evrogen, Moscow, Russia). In our study, only the reverse primers from each pair were applied for sequencing. The PCR program consisted of an initial denaturation at 94°C for 3 min, followed by 30 cycles of denaturation at 95°C for 30 s, annealing at 49°C for 30 s, and extension at 72°C for 90 s. Sequencing was performed using the facilities of the Core Centrum “Genomic Technologies, Proteomics and Cell Biology” (ARRIAM, Saint Petersburg, Russia) and Evrogen (Moscow, Russia).

### Physiological and biochemical assays

Enzymatic activity and substrate utilization were evaluated using the GEN III MicroPlate system (Biolog Inc., USA), which tests the ability of bacteria to metabolize 71 carbon sources and to grow in the presence of 23 inhibitory compounds (56–58). Fresh overnight cultures were processed according to the manufacturer’s instructions. Colonies were collected with a sterile cotton swab, suspended in 10 mL of inoculating fluid (IF-A), and adjusted to 96%–98% transmittance (T90) using a Biolog turbidimeter. Aliquots (100 μL) of the suspension were dispensed into each well of GEN III microplates with a multichannel pipettor. Plates were incubated at 27°C for 24 h (BINDER, Germany), and results were recorded using a MicroStation reader (Biolog Inc., USA).

Catalase and urease activities were determined with commercial diagnostic kits (NICF, Russia) following the manufacturer’s instructions. All assays were performed in duplicate.

### Chemotaxonomic characterization

Cellular fatty acid (FA) composition was determined, as described previously (59, 60), with minor modifications. Briefly, cells were harvested by centrifugation, disrupted in a glass–glass homogenizer, and extracted with chloroform–methanol (2:1, v/v) (61). The chloroform phase was collected and evaporated under an argon stream. Lipid extracts were transmethylated by incubation in anhydrous methanol containing 2% (v/v) H₂SO₄ at 80°C for 1.5 h under argon with continuous mixing. Heptadecanoic acid (C17:0) (Fluka, Buchs, Switzerland) was used as an internal standard. The resulting fatty acid methyl esters were analyzed by gas chromatography (59). Separation and identification were based on a comparison with retention times of reference standards (Sigma, St. Louis, MO, USA) and by analysis of mass spectra using an Agilent 7890 gas chromatograph equipped with a 30-m HP5MS UI capillary column and an Agilent 5970 mass-selective detector (Agilent, Santa Clara, CA, USA).

### DNA extraction and library preparation

Genomic DNA for Oxford Nanopore sequencing was isolated using the MagBeads FastDNA Kit for Microbiome (MP Biomedicals, Santa Ana, CA, USA) according to the manufacturer’s instructions. Libraries were prepared following the manufacturer’s protocol with the Native Barcoding Kit 24 V14 (Oxford Nanopore Technologies Ltd, Oxford, UK), the NEBNext Companion Module for Oxford Nanopore Technologies Ligation Sequencing (New England Biolabs, Ipswich, MA, USA), NEBNext Quick Ligation Reaction Buffer, and Blunt/TA Ligase Master Mix (New England Biolabs). Sequencing was performed on a MinION Mk1B platform (Oxford Nanopore Technologies Ltd) using an R10 flow cell.

DNA for Illumina NovaSeq X (Illumina Inc., San Diego, CA, USA) sequencing was extracted and processed, as described previously (62), with modifications. Briefly, cells were grown overnight in liquid Spizizen medium (per liter: (NH₄)₂SO₄, 2 g; KH₂PO₄, 6 g; Na₃C₆H₅O₇, 1 g; MgSO₄·7H₂O, 0.2 g; glucose, 5 g; K₂HPO₄·3H₂O, 18.3 g; tryptone, 20 g; yeast extract, 5 g) (63, 64), harvested, and washed with buffer (0.01 M EDTA, 0.15 M NaCl, pH 8.0). The suspension was incubated at 37°C for 60 min with ribonuclease A (10 mg/mL), lysozyme (20 mg/mL), and mutanolysin (1 mg/mL). Proteinase K (600 U/mL) was then added for 10 min at 37°C, followed by treatment with 10% sodium dodecyl sulfate for 10 min at 65°C. Instead of phenol–chloroform extraction, Protein Precipitation Solution (Qiagen, Venlo, Netherlands) was used. Subsequent precipitation and quality control steps were performed as previously described.

### Obtaining the annotated whole genome assembly

We employed long reads using the Oxford Nanopore platform and 2 × 100 bp short reads sequenced with Illumina NovoSeq X by Novogene Co., Ltd. in Beijing, China. Processing and quality assurance procedures of the long reads were carried out with RabbitQCPlus v2.2.9 (65), whereas short reads were processed and inspected with fastp v0.23.2 (66) and FastQC v0.12.1 (67). The initial genome assembly was generated with Flye v2.9.5-b1801 (68) software using long reads obtained with an R10 flow cell and basecalled with Dorado v0.8.3. (https://github.com/nanoporetech/dorado). Next, two polishing procedures were applied, namely, long reads-based consensus reconstruction with Medaka v2.0.1 (Oxford Nanopore Technologies Ltd, UK) and polishing with Illumina reads using BWA 0.7.18-r1243-dirty (69), SAMtools v1.21-21-gaa8cb59 (70), and Pilon v1.24 (71). Gene prediction and annotation were performed with Prokka v1.14.6 (72). To characterize the quality of the obtained genome, we applied CheckM v1.2.2 (73), BUSCO v5.4.2 (20), and QUAST v5.2.0 (74). For further functional characterization, we detected BGCs (Biosynthetic Gene Clusters) with antiSMASH v7.1.1 (75) and genome regions susceptible to HGT (Horizontal Gene Transfer) using Alien_hunter v1.7 (21). To retrieve the gene-to-gene comparison map of BGCs, we employed the suspected streptozotocin-encoding cluster, the reference BGC from the MIBiG database (accessed 18 January 2026) (76), and the closest genomic region relative to the strain nc5.1^T^ from a genomic data set. Graphical syntenic representation of the loci-wise comparisons was obtained with clinker v0.0.32 (77). To analyze sporulation-controlling genes, we collected all protein sequences proven to regulate the major sporulation stages in *Bacillus subtilis* from the SubtiWiki resource (accessed 25 December 205) (22). Homologs in the reference type strains, including nc5.1^T^, were mined using the MMseqs2 v.14.7 utility (78) and picked hits with the best identity estimates relative to reference proteins.

### Comparative taxonomic analysis of the nc5.1 strain with the closest species

The data set for taxonomic analysis was based on all assemblies of the *Bacillales* order deposited in the NCBI RefSeq (10) resource. We employed the average nucleotide identity (ANI)-based approach to calculate the pair-wise similarity with fastANI v1.33 (79) and next selected the closest assemblies of the *Psychrobacillus* genus. The metadata were obtained from the NCBI BioSample database (80). Genomes were then compared based on the digital DNA-DNA hybridization (DDH) metrics with the GGDC resource (81). To retrieve 16S rRNA sequences, we utilized Barrnap v0.9 (https://github.com/tseemann/barrnap), followed by the maximum likelihood (ML) tree reconstruction with RAxML-NG v1.2.2 (82). The whole genome-based phylogeny was built on the pangenomic inferences. Pangenome reconstruction was carried out with Panaroo v1.5.0 (83) with subsequent alignment of the core genes using MAFFT v7.429 (84) and single nucleotide polymorphism (SNP) extraction from the concatenated alignment with the SNP-sites v2.5.1 (85) utility. The best evolutionary model was determined using ModelTest-NG v0.1.7 (86), followed by the ML tree reconstruction with RAxML-NG v1.2.2 (82). The plots and trees were visualized with R v4.5.0, implementing the ggplot2 v3.3.5 (87) and ggtree v1.16.6 (88) packages. Additionally, we compared the phylogenies topologically using tqDist v1.0.2 (89).

### Cytotoxicity assay

The cytotoxic activity of *P. syltrankelensis* nc5.1^T^ culture supernatants against the human pancreatic cancer cell line PANC-1 (ATCC CRL-1469) was evaluated using the MTT assay. The strain was grown in liquid 2YT medium at 28°C with shaking at 190 rpm. Aliquots were collected after 13, 26, and 44 h of cultivation, and cell-free supernatants were obtained by filtration through 0.45-µm PVDF syringe filters. PANC-1 cells were seeded into 96-well plates (90 µL per well) in DMEM (high glucose) without phenol red (Pricella, Elabscience, Wuhan, China) supplemented with 10% FBS, 100 U/mL penicillin, and 100 µg/mL streptomycin (Capricorn Scientific, Germany). Filtered supernatants were added to the cultures at final dilutions of 1:10, 1:100, and 1:1,000. To exclude potential non-cellular reduction of MTT by bacterial metabolites, parallel control wells containing complete medium supplemented with the respective supernatant dilutions but without PANC-1 cells were included for each condition. Cells were incubated at 37 °C in 5% CO₂ for 24 h.

Cell viability was determined according to Kumar et al. (90). Briefly, 10 µL of MTT solution in PBS (5 mg/mL) was added to each well, followed by incubation for 4 h at 37°C in 5% CO₂. Then, 100 µL of SDS–HCl solution (10% SDS, 0.01 N HCl) was added, and the plates were incubated for an additional 18 h. Absorbance was measured at 570 nm and corrected for background at 620 nm.

All experiments were performed in seven replicates. Statistical analysis was carried out using one-way analysis of variance (ANOVA), followed by the emmeans post hoc test (emmeans R package v1.10.7; https://github.com/rvlenth/emmeans). A *P*-value < 0.05 was considered significant. Data were normalized to the median value of the control group, and all analyses were conducted in R v4.4.3 (91).

The human pancreatic carcinoma cell line PANC-1 was obtained from the shared research facility “Vertebrate cell culture collection” of the Institute of Cytology of the Russian Academy of Sciences (St. Petersburg, Russia).

## Data Availability

The genomic data of the P. syltrankelensis nc5.1^T^ are available in the NCBI databases, namely GenBank assembly GCA_055454035.1, BioProject (PRJNA1306386), BioSample (SAMN50633555), and SRA (SRS26222033).
